# Tuberculous meningitis: presentation, diagnosis and outcome in hiv-infected patients at the douala general hospital, cameroon: a cross sectional study

**DOI:** 10.1186/1742-6405-10-16

**Published:** 2013-06-11

**Authors:** Henry Namme Luma, Benjamin Clet Nguenkam Tchaleu, Bertrand Hugo Mbatchou Ngahane, Elvis Temfack, Marie Solange Doualla, Marie Patrice Halle, Henry Achu Joko, Sinata Koulla-Shiro

**Affiliations:** 1 Internal Medicine Unit, Douala General Hospital, Douala, Cameroon; 2 Faculty of Medicine and Biomedical Sciences, University of Yaoundé 1, Yaoundé, Cameroon; 3 Universite des Montagnes, Bagangté, Cameroon; 4 Faculty of Medicine and Pharmaceutical Sciences, University of Douala, Douala, Cameroon

**Keywords:** Tuberculous meningitis, Cerebrospinal fluid, Hydrocephalus, HIV

## Abstract

**Introduction:**

Tuberculous meningitis (TBM) the most fatal presentation of tuberculosis (TB) especially in HIV-infected patients is a real diagnostic and therapeutic challenge worldwide. In Cameroon where HIV and TB are amongst the leading public health problems, the magnitude of TBM has not been defined. Therefore, the objective of this cross sectional study was to describe the presentation and in-hospital outcome of TBM among HIV patients in Douala as well as its diagnostic difficulties.

**Methods:**

We did a clinical case note analysis of all HIV-1 infected patients treated for TBM in the Internal medicine unit of the Douala General Hospital, between January 1^st^ 2004 and December 31^st^ 2009. The diagnosis of TBM was made using clinical, laboratory [cerebrospinal fluid (CSF) analysis] and/or brain computerised tomographic (CT) scan features.

**Results:**

During the study period, 8% (54/672) of HIV-infected patients had TBM. Their mean age was 40.3 ± 12.7 years. The main presenting complaint was headache in 74.1% (40/54) of patients. Their median CD4 cell count was 16 cells/mm^3^ (IQR: 10 – 34). CSF analysis showed median protein levels of 1.7 g/l (IQR: 1.3 – 2.2), median glucose level of 0.4 g/l (IQR: 0.3 – 0.5) and median white cell count (WCC) count of 21 cells/ml (IQR: 12 – 45) of which mononuclear cells were predominant in 74% of CSF. Acid fast bacilli were found in 1.9% (1/54) of CSF samples. On CT scan hydrocephalus was the main finding in 70.6% (24/34) of patients. In hospital case fatality was 79.6% (43/54).

**Conclusion:**

TBM is a common complication in HIV-infected patients in Douala with high case fatality. Its presumptive diagnosis reposes mostly on CSF analysis, so clinicians caring for HIV patients should not hesitate to do lumbar taps in the presence of symptoms of central nervous system disease.

## Background

Tuberculous meningitis (TBM) is the most severe and dreaded manifestation of tuberculosis (TB) [1,2]. The HIV pandemic which predominates in low resource settings [3] has escalated the burden of TBM most especially as HIV disease progression increases the risk of extra-pulmonary tuberculosis [4]. In Cameroon, the burden of HIV is estimated at 5.3% of the adult population [3] and the annual incidence of TB estimated at 177 per 100 000 people per year [5] with HIV-TB coinfection rates approaching 50% [6,7]. Evidence shows that HIV-infected individuals are at greater risk of developing TBM, particularly at more advanced stages of immune-depression [8], with mortality rates as high as 67% against 25% in HIV uninfected individuals [2,9,10]. Despite treatment, mortality and long-term disability still remains unacceptably high thereby iterating that prevention, early recognition, diagnosis and appropriate treatment are fundamentals to improving outcomes [11]. However, the diagnosis and treatment of TBM still remains difficult irrespective of setting and becomes even more challenging when patients are HIV co-infected [2]. Pathologically, TBM usually results from haematogenous spread of primary or post primary pulmonary infection or from the rupture of sub-ependymal tubercle into the subarachnoid space [12]. Meningeal involvement in this case mostly is non-specific making suspicion and diagnosis difficult. Therefore, in order to optimize medical care for TBM, and to raise awareness, descriptive studies in different settings are useful for a better understanding of the impact of this disease and also to determine possible changes in its presentation over time [12,13]. In Cameroon, such studies are rare in spite of the high burden of both HIV [3] and tuberculosis [5]. More so, with the low scale up and low access to antiretroviral therapy [14], most HIV-infected patients still present with severe immune depression thus a higher risk of TBM. In this light, we decided to describe the clinical and radiological features of HIV-infected patients admitted to the Douala General Hospital with a presumed diagnosis of TBM, their outcome on treatment as well as the diagnostic difficulties associated with TBM.

## Methods

### Study setting, patients and diagnosis of TBM and HIV

After local institutional ethical approval, we carried out a cross sectional study at the Douala General Hospital, a specialist hospital in Douala, Cameroon. This hospital with a capacity of 320 beds is the main referral hospital in the sub region. We included in the study all adult (age >18 years) HIV-1-infected patients admitted to the internal medicine unit of this institution between January 1^st^ 2004 and December 31^st^ 2009 and treated for presumed TBM. Identification of eligible patients for the study was done by using the hospitalisation register of the Internal Medicine unit. From this register which contains patients’ diagnoses and outcome, we sorted those with presumed TBM and obtained their files from the archives. Socio-demographic and other information relevant to the study were obtained from the files and recorded in a case reporting form (CRF). Information on antiretroviral therapy (ART) was not collected because very few patients were on ART during the study period which represented the era when ART had to be paid for by patients, most of whom could not afford because of its high cost. In this hospital, diagnosis of TBM is presumed when a patient has clinical, biochemical and radiological features suspicious of extrapulmonary TB with meningeal involvement. Clinically, symptoms include fever, headache, seizure and on examination focal signs or neck stiffness, and/or seizures and/or altered mentation. In this institution, the diagnosis of CNS disease in HIV follows an algorithm whereby in the presence of CNS symptoms, a brain computerised tomographic (CT) scan is advised to exclude space occupying lesions and/or signs of raised intracranial pressure (ICP) before performing a lumbar tap (LP) for cerebrospinal fluid (CSF) analysis: protein levels, glucose levels and white cell count (WCC). Based on CSF analysis, TBM is presumed in the presence of high proteins, low glucose, high white cell count, absence of Cryptococcus (on Indian ink staining) and absence of bacteria that commonly cause meningitis (on gram stain) or their antigens (*Streptococcus pneumonia*, *Neisseria meningitis*, *Haemophilus influenzae*). TBM is confirmed when *Mycobacterium tuberculosis* is found in the CSF. A chest X-ray is also done in search of lung lesions suspicious of TB and three consecutive sputum samples on different days are collected for examination by Ziehl-Nielsen staining, in case there is a history of cough. For each patient, the decision to treat is based on a combination of clinical, radiological and biochemical argument or persistent or deteriorating clinical state during conventional treatment of bacterial meningitis. TB treatment is done with combination therapy of rifampicin, isoniazid, pyrazinamide and ethambutol for two months, then relay with rifampicin and isoniazid for six to eight months according to national guidelines [15]. Adjuvant to this treatment is corticosteroid, given to patients with deteriorating clinical states. HIV diagnosis at the Douala General Hospital is according to national guidelines [16] by antibody detection on two successive samples using a third generation ELISA test BIOREX® (Biorex Diagnostics Limited, Antrim, United Kingdom). When both samples are positive, a third sample is collected and tested using Genie® III HIV-1/HIV-2 Assay (Bio-Rad Diagnostics, Marnes la Coquette, France) to specify either HIV 1 or HIV 2. Patient is declared positive for HIV if these three tests are positive and if any discordance, testing is done using Western blot (New LAV blot, Diagnostics, Pasteur, Marnes la Coquette, France).

### Statistical analysis

The data collected was analysed using STATA 11.2 statistical software (Stata Corporation, College Station, Texas). The main outcome of interest was in-hospital mortality. Clinical features were categorised as either present or absent. Based on the British Medical Research Council (BMRC) TBM grading system [17] but slightly different because the only criterion we used was focal signs (consciousness not assessed), patients were graded as grade I (absence of focal signs) and grade II/III (presence of focal signs). Continuous variables were expressed using means and standard deviations or medians and interquartile range (IQR) where necessary. For comparison, continuous variables were later categorised with defined cut-off values. Results were presented in tables most of which were expressed as percentages of the study population. Given the low values in most cells of the tables, comparison was done using Fisher’s exact test. Logistic regression was attempted, but a final model was not built because the small sample size rendered some cells of contingency tables empty, thus making this analysis impossible. Evidence of association was considered for a two-tailed p-value <0.05.

## Results

### Characteristics of study population

During the study period, 672 files of HIV infected patients were studied, of which 54 were retained for the study, giving a prevalence of presumed TBM of 8%. The study population was predominantly male (Table 1). The mean age of the study population was 40.3 ± 12.7 years. The most common symptom was headache (Table 2). The median CD4 cell count of the patients was 16 cells/mm^3^ (IQR: 10 – 34). Men had lower median CD4 cell count than women: 14 cells/mm^3^ (IQR: 9 – 19) vs. 27 cells/mm^3^ (IQR: 12 – 50). All patients had a lumbar tap (LP) done for cerebrospinal fluid (CSF) analysis and pre-LP CT scan was done in 62.9% (34/54) of patients. The main CT scan findings are shown on Table 2. Macroscopically, 64.8% (35/54) of CSF were clear. CSF analyses showed a median protein level of 1.7 g/l (IQR: 1.3 – 2.2), median glucose level of 0.4g/l (IQR: 0.3 – 0.5) and median white cell count (WCC) count of 21 elements/ml (IQR: 12 – 45) with predominantly mononuclear cells in 74% of samples (Table 2). *Mycobacterium tuberculosis* was identified in 1.9% (1/54) of samples after Ziehl Nielson stain (Table 2). CSF mycobacterial culture was not done due to logistic reasons.

**Table 1:** General characteristics of 54 patients presumptively diagnosed with CNS tuberculosis

**Characteristics**	**N (%)**
Age group	
<30	10 (18.5)
30 – 39	19 (35.2)
40 – 49	8 (14.8)
50 – 59	14 (25.9)
>60	3 (5.6)
Sex	
Male	30 (55.6)
Female	24 (44.4)
Marital Status	
Single	11 (20.4)
Married	27 (50.0)
Divorced	2 (3.7)
Widow(er)	14 (25.9)
CD4 cell groups	
<50	47 (87.0)
50 – 200	4 (7.4)
>200	3 (5.6)

**Table 2:** Clinical, laboratory and radiological findings among patients with presumed TBM

**Findings**	**n**	**% (n/N)**
Clinical presentation (N=54)		
Headache	40	74.1
Fever	32	59.3
Neck stiffness	31	57.4
Focal signs	22	40.7
CSF findings (N=54)		
*Glucose <0.6 g/l	47	87.0
Proteins >0.5 g/l	54	100
White cell count (WCC) >5/μL	51	94.4
Predominantly mononuclear cells in total WCC	40	74.0
Positive Smear in CSF	1	1.9
CT scan findings (N=34)		
Hydrocephalus	24	70.6
Meningeal contrast enhancement	16	47.1
Cortical atrophy	4	11.8
Chest X-ray (N=54)		
Suspicious of TB	24	44.4

*Normal glucose in CSF considered to be 2/3 serum glucose averagely 1 g/l (0.7 - 1.15).

### Grading and outcome of CNS TB patients

Based on the presence of focal signs, 59.3% (32/54) of our patients were grade I (no focal signs) and 40.7% were grade II/III. The case fatality rate in our study population was 79.6% (43/54), 51.2% (22/43) of whom were grade II/III. Of the total deaths, male sex represented 69.8% (30/43). The median CD4 cell count of patients who died was lower than that of those who survived: 16 cells/mm^3^ (IQR: 10 – 31) vs. 20 cells/mm^3^ (IQR: 11 – 209), p<0.001. There were no statistically significant differences between the CSF findings of patients who died and those who survived. Older age, headache and neck stiffness were not associated with death (Table 3).

**Table 3:** Contingency table comparing those who died to those who survived among 54 patients with TBM

	**Died**	**Survived**	**P-value (Fisher’s exact)**
Age >40years			
Yes	21	4	0.5
No	22	7	
Sex			
Male	30	0	0.001
Female	13	11	
Headache			
Yes	33	4	0.4
No	10	7	
Neck stiffness			
Yes	24	7	0.7
No	19	4	
Focal signs			
Yes	22	0	0.002
No	21	11	
Hydrocephalus			
Yes	20	4	0.01
No	4	6	

## Discussion

Though TBM is among the first three causes of CNS opportunistic infections in HIV/AIDS patients in sub-Saharan Africa, South America and Asia (the other two being cerebral toxoplasmosis and cryptococcal meningitis) [18,19] its real incidence and prevalence are not well known in these resource limited settings [20]. This study which had as one of its aims bringing to light the diagnostic difficulties of TBM in Cameroon showed that the in-patient prevalence of TBM in HIV-1 at the Douala General Hospital during the study period was 8% (54/672). This finding though similar to that of an Indian study [20] which found TBM in 7% of 375 patients with HIV, we think it grossly underestimates the prevalence of TBM in our setting. Irrefutably, one of the main problems facing the unavailability of data on the burden of TBM around the world is the challenge associated with its confirmatory diagnosis. As with other forms of TB, the gold standard of diagnosis is isolation of tuberculous bacilli through detection by acid fast staining and/or culture of CSF. Nevertheless, the yield of acid fast staining and culture still remains low [20,21]. This fact could be illustrated in our study where confirmatory diagnosis by bacillary microscopy was in only 1.9% (1/54) of patients, the rest of the diagnosis being presumptive. This, however, is not surprising given the low sensitivity of acid-fast smear in CSF on the order of 20 – 40% [22] and the fact that this sensitivity greatly depends on the expertise of the laboratory personnel and the frequency of LPs as well as the volume collected. In one study, collecting 10 ml of CSF and centrifuging increased the sensitivity of acid-fast bacilli yield to 69% [23]. Given that we usually collected 1 – 2 ml could explain why we had such low detection rates. Some other studies had similarly low detection rates [10,13,24]. Therefore, for more yield from acid fast microscopy, a minimum of 5 ml of CSF should be collected [2] and centrifuged [23] so as to increase sensitivity, this especially in a low income setting like ours. On the other hand, acid fast bacilli culture is not our routine practice due to logistic reasons and the long delay for availability of results. In our setting therefore, most diagnoses are based either on clinical suspicion (and an associated favourable response to empirical treatment) or on indirect clinical, laboratory and/or radiological features suspicious of TBM, the most crucial of which is CSF analysis [8].

Clinical features were useful in orientating clinical suspicion towards CNS disease, though they usually were non-specific and not outstanding. In our study, headaches and fever were the most common symptoms involving 74.1% and 59.3% of our patients respectively. Though these finding were similar to those of some studies [1,2,25] they were less than the over 80% prevalence in other studies [26,27]. This discrepancy could be simply because our patients were more severely immune depressed and patients with low CD4 counts are more likely to present atypically [21]. More so, classical features expected in bacterial meningitis were uncommon in our TBM patients [28] probably because TBM being a sub-acute disease with insidious onset and variable duration of symptoms from a few days to many months [29] there is possibility of symptom modification by previous non-specific treatment as is often the case with many patients we receive in our referral institution. However once suspected, initiation of anti-TB treatment becomes urgent because of the associated high case fatality.

We therefore suspected TBM in the presence of CNS disease orientating symptoms/signs and the diagnosis relied almost entirely on CSF finding of high proteins levels, low glucose levels and leucocyte pleocytosis especially where there was mononuclear cell predominance [2,8,28]. We also used CT scan features. In CSF we found that the median WCC in our study was similar to that of one study [25] but was however lower than that of others [1,2,8,26]. It is known that CSF WCC is lower in HIV-infected TBM patients compared to those who are HIV-uninfected [8] and patients with advanced HIV disease usually have low numbers of lymphocytes in the peripheral blood, which may be reflected by a low lymphocyte count in the CSF. Moreover, TBM may stimulate increased HIV replication in the CNS resulting in destruction of CSF lymphocytes [21] this especially as our study population was made up of severely immune depressed patients. Therefore in settings like ours where the burden of both TB and HIV overlap [3,30], LPs for CSF analysis should be part of HIV patients care package in those with CNS symptom as early diagnosis and treatment of suspected TBM could be lifesaving.

CT scan findings in our TBM patients were mostly hydrocephalus and meningeal enhancement. The former, seen in 70.6% of our patients is similar to that in one study [31] but much higher than what was found in two other studies [25,27]. Given that in our institution, CT scan imaging of the brain is a common practice in CNS disease investigation, these findings in our patients raised much suspicion for TBM and together with CSF findings improved our presumption of TBM diagnosis. However, a remarkable proportion of our patients with hydrocephalus died in the course of TBM treatment. Given that the development of hydrocephalus either at presentation or in the course of TBM is a factor associated with mortality [32], our management of hydrocephalus which was neither medically nor neurosurgically [33] aggressive, could explain why most of our patients with hydrocephalus died.

TBM is the most deadly form of TB particularly in patients co-infected with HIV and severely immune depressed [8]; 79.6% (43/54) of our patients died a case fatality similar to that of other studies [9,34]. Though we found such high mortality, the real mortality is probably higher because some patients might have died before the diagnosis. In our study, most of those who died were men probably because they were more profoundly immune depressed than women as reflected by their median CD4 count. Also, the presence of focal signs and altered consciousness on admission, were found in other studies to be strongly associated with death [2]. In our study, all those who presented with focal signs died. According to the staging of the severity of TBM proposed by the British Medical Research Council (BMRC) Streptomycin in tuberculosis trial committee [18], the presence of focal signs is a factor associated with poor prognosis and this could be reflected in our study whereby majority of the deaths were among those graded BMRC grade II/III.

There were several limitations in our study. First of all, its retrospective nature and the fact that information was abstracted from medical case notes, (which often are not uniformly recorded) created the problem of missing data and probably resulted in a remarkable proportion of eligible patients not included in the study. Secondly the small sample size limited our statistical analysis. Finally, being a hospital based study in a referral hospital in an urban area, it does not capture the real picture of the burden of TBM among HIV-infected patients in Cameroon. However, the study portrays the challenges faced by clinicians in the diagnosis and management of TBM in HIV especially in a setting with limited resources. With these challenges, we have designed and put in place, a prospective study with the aim of throwing more light on the burden of TBM both in HIV-infected and HIV-uninfected patients in Douala, from which we hope to obtain more robust data.

## Conclusion

TBM is a common CNS complication in HIV-infected patients in Douala. The definitive diagnosis of TBM is very difficult especially in resource limited settings. This diagnosis reposes mostly on analysis of sufficient volumes (at least 5 ml) of CSF, as such, in the presence of any CNS symptoms, clinicians caring for HIV patients should not hesitate to do lumbar taps because these symptoms may be revelatory of TBM, a complication with very high case fatality. Treatment of TBM, should also take into consideration specific management of its complications, amongst which hydrocephalus is the most outstanding.

## Competing interest

The authors of this piece of work declare no conflict of interest.

## Authors’ contribution

HNL, BCNT and ET designed the study. BCNT, BHNM and MPH collected the data and together with ET, they entered the data. ET and HNL analysed the data and were assisted by BCNT and MSD in the manuscript write up. HAJ, SKS, MSD and MPH did the manuscript proof-reading and editing. All authors read and approved the final manuscript to be submitted.
